# Differences in Uniquely Identified Peptides Between ddaPASEF and diaPASEF

**DOI:** 10.3390/cells13221848

**Published:** 2024-11-07

**Authors:** Mio Iwasaki, Rika Nishimura, Tatsuya Yamakawa, Yousuke Miyamoto, Tsuyoshi Tabata, Megumi Narita

**Affiliations:** 1 Center for iPS Cell Research and Application, Kyoto University, Kyoto 606-8507, Japan; rika.nishimura@cira.kyoto-u.ac.jp (R.N.); yamakawa@cira.kyoto-u.ac.jp (T.Y.); kumazaki@cira.kyoto-u.ac.jp (M.N.); 2 MassSoft, Sakyo-ku, Kyoto 606-8501, Japan

**Keywords:** DDA, data-dependent acquisition, DIA, data-independent acquisition, nanoLC-MS/MS, phospho-proteome, ubiquitin–proteome

## Abstract

Recent advancements in mass spectrometry-based proteomics have made it possible to conduct comprehensive protein analysis. In particular, the emergence of the data-independent acquisition (DIA) method powered by machine learning has significantly improved protein identification efficiency. However, compared with the conventional data-dependent acquisition (DDA) method, the degree to which peptides are uniquely identified by DIA and DDA has not been thoroughly examined. In this study, we identified over 10,000 proteins using the DDA and DIA methods and analyzed the characteristics of unique peptides identified by each method. Results showed that the number of peptides uniquely identified by DDA and DIA using the same column type was 19% and 32%, respectively, with shorter peptides preferentially detected by the DIA method. In addition, more DIA-specific peptides were identified, especially during the first 10% of elution time, and the overall 1/*K*_0_ and *m*/*z* shifted toward smaller values than in the DDA method. Furthermore, comparing the phosphorylation and ubiquitination proteome profiles with those of whole-cell lysates by DDA showed that the enrichment of post-translationally modified peptides resulted in wider *m*/*z* and 1/*K*_0_ ranges. Notably, the ubiquitin peptide-enriched samples displayed lower *m*/*z* values than the phospho-proteome. These findings suggest a bias in the types of peptides identified by the acquisition method and the importance of setting appropriate ranges for DIA based on the post-translational modification of peptide characteristics.

## 1. Introduction

Recent developments in mass spectrometry-based proteomics technologies have enabled comprehensive protein identification. In particular, the introduction of data-independent acquisition (DIA) methods has improved identification efficiency by approximately a factor of two over data-dependent acquisition (DDA), allowing the identification of over 10,000 human proteins in a single analysis [1,2,3,4]. The DDA method is a conventional proteome analysis method that obtains a set of MS/MS fragment ions corresponding to each precursor ion and uses this information in a database search to identify peptides from the precursor ion mass with the amino acid information obtained from the fragment ions. In contrast, the DIA method continuously acquires MS/MS fragment ions over the entire mass range while selecting a wide range of *m/z* values (10–40 *m/z*) [5,6]. Multiple co-isolated peptide ions over a wide *m/z* range typically generate complex MS/MS fragment ions. Modern DIA analysis software such as DIA-NN and Spectronaut deconvolutes these spectra to identify various peptides, usually compared to a previously acquired peptide library; however, machine learning-powered *in silico*-predicted libraries have often been used recently [7], thus enabling direct DIA and facilitated proteome analysis. However, there are some critical issues with the current DIA methods. It is still challenging to use *in silico*-predicted libraries for post-translational modifications because of their relatively low sensitivity [8]. In addition, while DIA represents the entirety of peptides identifiable by DDA, some peptide ions are uniquely identified using each method. Although there have been studies on the degree to which the accuracy of quantification differs between DIA and DDA [9,10], the characteristics of each identified peptide have not been examined extensively.

In this study, we analyzed the characteristics of peptides uniquely identified by DDA and DIA by maximizing peptide identification using a single MS/MS analysis. The resulting elution profiles showed that peptides were very crowded, particularly during the first half of elution time, and their contribution to all identified peptides was also high. Furthermore, phosphorylated- and ubiquitinated-peptide analyses showed distinct features in optimal *m/z* and 1/*K*_0_ ranges distinct from whole proteome analysis, indicating the need to match the characteristics of each post-translational modification in the DIA analysis settings.

## 2. Materials and Methods

### 2.1. Materials

Acetonitrile (#018-19853), trifluoroacetic acid (TFA, #204-02743), sodium deoxycholate (SDC, #190-08313), sodium lauroyl sarcosinate (SLS, #192-10382), mass spectrometry-grade Trypsin with lysyl-endoprotease (Trypsin/Lys-C Mix; #V5072), dithiothreitol (DTT, #045-08974), iodoacetamide (IAA, #095-02151), ammonium bicarbonate (#018-21742), and formic acid (FA, #067-04531) were purchased from Wako. Y-27632 (#Y0503), protease inhibitor (#P8340-1ML), phosphatase inhibitor cocktail 2 (#P5726-1ML), phosphatase inhibitor cocktail 3 (#P0044-1ML), and tris(2-carboxyethyl)phosphine (TCEP, C4706-2G) were purchased from Sigma-Aldrich. HeLa Protein Digest Standard (#88328), TrypLE Select (#12563011), the Pierce BCA Protein Assay Kit (#23225), and the Quantitative Peptide Assay Kit (Cat#23290) were purchased from Thermo Fisher Scientific (Waltham, MA, USA). D-PBS (#13249-24, Nacalai Tesque, Kyoto, Japan), SDB-XC Empore disc cartridges (#2340, 3M, MA, USA), 1M Tris-HCl (pH 9.0) (#314-90381, Nippon gene, Tokyo, Japan), laminin-511 E8 (#892012, Nippi, Tokyo, Japan), StemFit AK02N (AK02N, Ajinomoto, Tokyo, Japan), Ultrapure water (#11307-79, KANTO KAGAKU, Tokyo, Japan), the PTMScan HS Ubiquitin/SUMO Remnant Motif (K-ε-GG) Kit (#59322, Cell Signaling Technology, Danvers, MA, USA), and Sera-Mag SpeedBead carboxylate-modified magnetic particles (hydrophilic particles, Cat# 45152105050250; hydrophobic particles, Cat# 65152105050250; Cytiva, Tokyo, Japan) were also used in this study.

### 2.2. Sample Preparation

Human induced pluripotent stem cells (hiPSCs; 692D2) were cultured in StemFit AK02N on iMatrix-511-coated dishes. Cells were lysed with ice-cold PTS buffer (100 mM Tris-HCl pH 9.0, 12 mM SLS, 12 mM SDC with 1% protease, and 1% phosphatase inhibitors) after washing once with ice-cold PBS. Lysates were used as protein samples and processed using a modified protein aggregation capture method [11]. Briefly, hydrophilic and hydrophobic Sera-Mag SpeedBead carboxylate-modified magnetic particles were mixed in equal parts and washed three times with distilled water. Beads were reconstituted in distilled water at 15 µg beads/µL. Protein samples were reduced with 20 mM TCEP (final conc.) and alkylated with 36 mM IAA (final concentration). Alkylated protein samples were mixed with 20 µL reconstituted beads and ethyl alcohol at a final concentration of 75% (*v/v*). Mixed samples were washed twice with 80% ethyl alcohol. After removing the supernatant, samples were resuspended in 100 µL of 50 mM Tris-HCl (pH 8.0) with 2 µL trypsin/Lys-C Mix at 37 °C overnight, for protein digestion. Digested samples were acidified with 20 µL of 5% TFA and desalted using an in-house SDB-XC StageTip [12], and peptide concentrations were measured using a quantitative peptide assay kit. For phosphopeptide enrichment, 100 μg of peptides measured by the BCA assay was used for HAMMOC, as previously reported [13]. The PTMScan HS Ubiquitin/SUMO Remnant Motif (K-ε-GG) Kit was used for ubiquitinated-peptide enrichment using 1080 µg of peptides measured by the BCA assay, according to the manufacturer’s instructions.

### 2.3. Nano-Liquid Chromatography (nanoLC)–Mass Spectrometry (MS/MS) Analysis

According to the gradient time length, separate portions of the lysed HeLa protein digest (Thermo Fisher Scientific, MA, USA) were loaded and separated using a nanoElute (Bruker, MA, USA) or Dionex UltiMate 3000 RSLCnano System (Thermo Fisher Scientific, MA, USA). For 90, 225, 450, and 600 min gradient analysis, 200, 500, 1000, and 1500 ng of HeLa protein digest were used, respectively. Four types of columns were used: an Aurora column (AUR3-25075C18-CSI, 250 mm length, 75 mm i.d., IonOpticks, MEL, AUS), a self-pulled capillary column with CAPCELL MP beads (2.8 μm beads, 250 mm length, 75 μm i.d., #51224, Osaka Soda, Osaka, Japan), ReproSil-Pur 120 C18-AQ beads (1.9 μm beads, 250 mm length, 75 μm i.d., #r119. Aq, Dr. Maisch, Ammerbuch-Entringen, DEU), or monolithic column (4000 mm length, 100 μm i.d., GL Science, Tokyo, Japan). The Dionex UltiMate 3000 RSLCnano System with an HTC-PAL autosampler (CTC Analytics, Zwingen, CHE) was used for the analysis with ReproSil-Pur 120 C18-AQ beads with a 450 min gradient time, with a C18 monolithic silica column for 90, 240, 450, and 600 min gradient time. Separated peptides were analyzed by ddaPASEF using a timsTOF Pro system (Bruker, Billerica, MA, USA). All diaPASEF analyses were performed using a timsTOF Pro2 system (Bruker, Billerica, MA, USA).

The mobile phase, comprising 0.1% formic acid (solution A) and 0.1% formic acid in acetonitrile (solution B), used a flow rate of 400 nL/min. For the 90 min analysis, 2–17% solution B was used for 60 min, 17–25% solution B for 30 min, and 25–37% solution B for 10 min. For the 225 min analysis, 2–17% solution B was used for 150 min, 17–25% solution B for 75 min, and 25–37% solution B for 25 min. For the 450 min analysis, 2–17% solution B was used for 300 min, 17–25% solution B for 150 min, and 25–37% solution B for 50 min. For the 600 min analysis, 2–17% solution B was used for 400 min, 17–25% solution B for 200 min, and 25–37% solution B for 67 min. The columns were washed with 80% solution B after the analysis. For the monolithic silica column, a longer washing step after 37%-to-80% solution B was used, because of its longer column length. Below is the number of measurements for each column used in this analysis.
**Column Types****90 min****225 min****480 min****600 min**Aurora*n =* 3*n =* 3*n =* 3*n =* 3Capcell*n =* 3*n =* 3*n =* 3*n =* 1Reprosil*n =* 3*n =* 3*n =* 3
Monolith*n =* 2*n =* 2*n =* 2*n =* 2Aurora DIA*n =* 3*n =* 3*n =* 3*n =* 3

The applied spray voltage was 1400 or 1500 V, and the interface heater temperature was 180 °C. For DDA analysis, the Parallel Accumulation Serial Fragmentation (PASEF) acquisition method [14] was used to obtain MS and MS/MS spectra. For ddaPASEF settings, 1.17 s per cycle with precursor ion scan and 10 PASEF scans were conducted with a precursor ion range of 100–1700 *m/z* and ion mobility ranges of 0.6–1.6 V·s·cm^−2^. For DIA analysis, the Parallel Accumulation Serial Fragmentation (PASEF) acquisition method with data-independent acquisition (DIA) mode was used (diaPASEF) [6] to obtain MS and MS/MS spectra. For diaPASEF settings, 1.74 s per cycle with precursor ion scan and 16 diaPASEF scans were conducted with an MS/MS isolation width of 28 *m/z*, precursor ion ranges of 391–1175 *m/z*, and ion mobility ranges of 0.69–1.39 V·s·cm^−2^.

### 2.4. Proteome Data Analysis for Protein Identification

Obtained DIA data were searched using DIA-NN (v1.9) [15] and Spectronaut v19.2 (Biognosys AG, Schlieren, CHE) against selected human entries of UniProt/Swiss-Prot release 2022_03 containing contaminant proteins [16] with cysteine carbamidomethylation as a fixed modification and protein N-terminal acetylation and methionine oxidation as variable modifications. For other DIA-NN and Spectronaut parameters, Trypsin protease, two missed cleavage, peptide-length range of 7–30, precursor *m/z* range of 300–1800, precursor charge range of 1–4, fragment-ion *m/z* range of 200–1800, and 1% precursor FDR were used.

DDA raw data files were processed using DataAnalysis software (v6.1, Bruker, MA, USA) to generate peak lists for DDA data of HeLa samples, which were analyzed using Mascot v2.5 (Matrix Science, London, UK), Comet [17], and X!Tandem [18], with fixed modifications of cysteine carbamidomethylation, variable modification of protein N-terminal acetylation and methionine oxidation against human entries of UniProt/Swiss-Prot release 2022_03 containing contaminant proteins [16] with a precursor mass tolerance of 20 ppm, a fragment-ion mass tolerance of 0.1 Da, and trypsin protease, and up to two missed cleavages allowed. Identified peptides were uniquely selected based on scan number, peptide sequence, and charge state. Peptides were rejected if the Percolator score (percolator-v3-04) [19] was above the 1% FDR cutoff of the spectrum peptide match with the result of the target and decoy database.

For the analysis of samples without any enrichment and enriched for phosphorylated and ubiquitinated peptides in hiPSCs (692D2), raw data files were directly processed using the PEAKS Studio 11.0 software (build 20230821, Bioinformatics Solutions Inc., Ontario, Canada) against human entries of UniProt/Swiss-Prot release 2024_02 containing contaminant proteins. Analysis parameters were the same as above, but serine, threonine, and tyrosine phosphorylation or lysin ubiquitination were added as a variable modification, with a precursor-mass tolerance of 15 ppm, a fragment-ion mass tolerance of 0.05 Da, and strict trypsin and Lys-C specificity, which allowed up to six missed cleavages.

The results output from different software programs cannot be combined, as is because of differences in the method of attribution of proteins and description of modification information. Therefore, to analyze the uniqueness of the DDA and DIA methods, the identified sequences were re-analyzed using the protein sequence database to obtain the protein information. Then, all identified peptides were grouped into protein groups based on previously established rules [20]. The post-translational modification information of the peptides was also modified using an in-house Perl script. To calculate the number of identified peptides, we excluded peptides with fewer than six amino acids and peptides listed in the contamination database. To calculate the number of identified proteins, we selected proteins with unique peptides, and did not include the number of grouped proteins. Finally, all data analysis steps were performed using an in-house Perl or R script. For gradient time analysis, the percent of elution time of identified peptides was calculated based on the total elution time of each gradient analysis. If different analyses identified the same peptide, the median percent of elution time was used for DDA or DIA analysis.

## 3. Results

### 3.1. Uniquely Identified Peptides Analyzed with DIA and DDA

**Figure 1:**
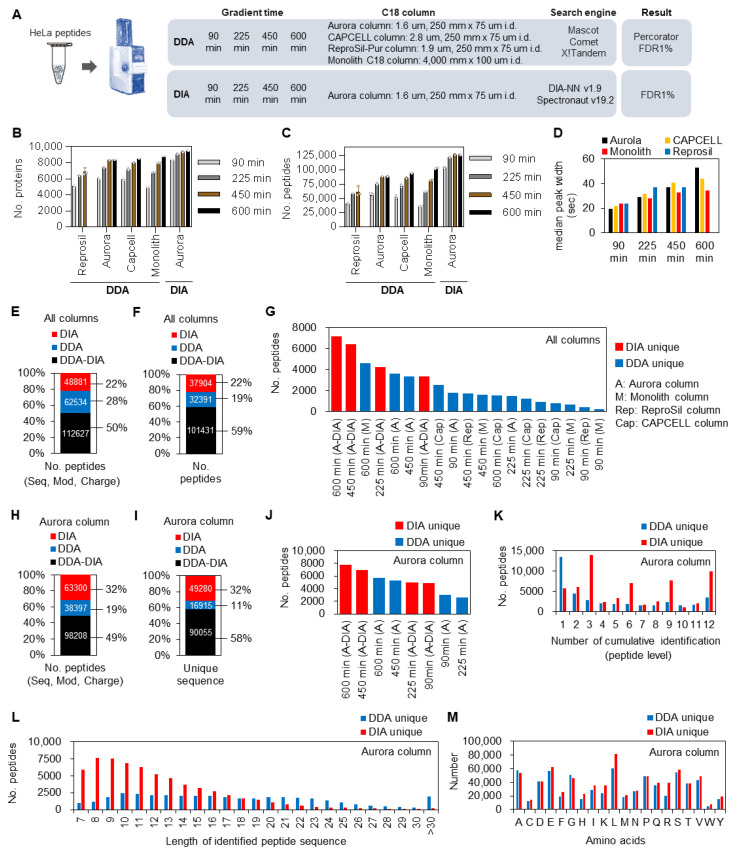
HeLa cell proteome analyzed by DDA and DIA methods. (**A**) Workflow for protein identification using DDA and DIA methods. Four gradient times and different column types were used for the DDA method, and four gradient times with an Aurora column were used for the DIA method. Three database search engines for DDA and two DIA software packages were used. See “Nano-liquid chromatography (nanoLC)-mass spectrometry (MS/MS) analysis” and “Proteome data analysis for protein identification” in the Materials and Methods section for details. (**B**) The number of identified proteins. See “Nano-liquid chromatography (nanoLC)–mass spectrometry (MS/MS) analysis” in the Materials and Methods section for the number of measurements for each column used in this analysis. (**C**) The number of identified peptides. (**D**) Median peak widths of peptides identified by each analytical column with gradient time. (**E**) The percentage and number of uniquely identified peptides by the DDA (*n =* 39) and DIA (*n =* 12) methods using all column types and gradient times. (**F**) The percentage and number of uniquely identified peptide sequences by the DDA (*n =* 39) and DIA (*n =* 12) methods using all column types and gradient times. (**G**) The number of uniquely identified peptides by each column and gradient time, using the same data as in (**E**). (**H**) The percentage and number of uniquely identified peptides by the DDA (*n =* 12) and DIA (*n =* 12) methods using the Aurora column with all gradient times. (**I**) The percentage and number of uniquely identified peptide sequences by the DDA (*n =* 12) and DIA (*n =* 12) methods using the Aurora column with all gradient times. (**J**) The number of uniquely identified peptides by each column and gradient time, using the same data as in (**H**). (**K**) The number of cumulative identifications of identified peptides by the DDA and DIA method, using the same data as in (**H**). (**L**) The length distribution of identified peptide sequences using the same data as in (**H**). (**M**) The distribution of the amino acid usage from the identified peptides by DDA and DIA methods, using the same data as in (**H**).

To determine which peptides were likely to be specifically identified by each analysis method, tryptic digests of HeLa cells were used as samples and measured by the DDA and DIA methods using four different columns and gradient times (Figure 1A). Multiple database search engines were used to collect data on the DDA and DIA methods to ensure maximal peptide identification. Although the number of identified proteins (Figure 1B, Table S1) and peptides (Figure 1C, Table S1) increased with increasing gradient time for all column types, the rate of increase differed, depending on the column type. In particular, the monolithic column identified the lowest number of peptides and proteins in the 90 min analysis, and these numbers increased markedly as the gradient time increased. Among DDA analyses, the highest peptide and protein identification numbers were observed in the 600 min analysis using a monolithic column, thus suggesting that longer column lengths with higher resolutions improve peptide identification efficiency over longer analysis times, consistent with a previous report [21]. Therefore, we compared the median width of identified peptide peaks with each column and gradient time (Figure 1D). While the Aurora column with the highest identification efficiency at 90 min analysis had the smallest peptide peak width, the monolithic column with the highest identification efficiency at 600 min analysis had the smallest peak width at 600 min. The monolithic column exhibited the smallest peak broadening with increasing gradient time. Because the number of peptides selected for MS/MS fragmentation per scan was limited by DDA, the sharpness of peptide peak has a significant advantage in terms of identification efficiency. In DIA, the identification efficiency was also improved by 1.1-fold by extending the gradient time, suggesting that the advantage of increasing the frequency of sharp selection of peptides by increasing separation time was also present in the DIA method.

Next, we analyzed the degree of overlap between measurement methods at the peptide (Figure 1E) and sequence levels (Figure 1F). The total number of identified unique proteins and peptides were 15,035 and 175,161 by DDA (*n =* 39) and 11,207 and 161,508 by DIA (*n =* 12), respectively. The results showed that most peptides were commonly identified by both methods, with 28% and 22% uniquely identified by DDA and DIA, respectively (Figure 1E). The percentage of DDA unique peptides decreases when peptide modification information is removed, indicating that the DDA method often identifies different modification patterns rather than sequence differences (Figure 1F). To keep conditions constant between DDA and DIA, we compared only the results obtained with the Aurora column, with the same gradient time, number of data, and injection volume (Figure 1H–M). The number of identified unique proteins and peptides were 12,056 and 136,605 by DDA (*n =* 12). Although the number of protein identifications was similar between DIA and DDA, the content of peptide types was 19% for DDA and 32% for DIA, with DIA having a higher percentage of unique peptide and sequence identifications (Figure 1H,I). We then analyzed the number of uniquely identified peptides according to column type and measurement time (Figure 1G,J). Notably, the DDA method paired with a short gradient time did not yield many unique peptides (i.e., most detected peptides were commonly identified). The 600 min analysis on the monolithic and Aurora columns showed the most uniquely identified peptides in the DDA method. In contrast, the peptides obtained by the DIA method also had more unique identification results, owing to their higher identification efficiency, with the 600 min analysis yielding the most uniquely identified peptides among all analyses.

Due to the stochastic nature of DDA MS/MS sampling, we calculated the number of times a peptide was uniquely identified by each method using the Aurora column (Figure 1K). The results indicated that many peptides were identified only once out of 12 measurements with DDA, suggesting that the cumulative number of identifications increased with DDA. By contrast, many peptides were identified in 3 or all 12 measurements by DIA, suggesting fewer missing values with this method. This property of DIA is an important advantage for quantitative analysis. Furthermore, when the lengths of peptide sequences uniquely identified by each method were compared (Figure 1L), while most DIA-specific peptides were shorter, DDA unique peptides showed a relatively broad distribution. In addition, there was no significant bias in amino acids in peptides uniquely identified by DDA and DIA (Figure 1M). These results indicate that not all the identification results obtained by the DDA method overlap with those obtained by the DIA method, and peptides uniquely identified by DDA and DIA were characterized by peptide sequence length and cumulative number of identifications.

### 3.2. Characteristics of Uniquely Identified Peptides Differed Between DDA and DIA

To understand why some peptides were uniquely identified by DDA and DIA and what makes them unique, we calculated the number of peptides and proteins uniquely identified by each method by elution time, mass-to-charge ratio (*m/z*), and reciprocal of reduced ion mobility (1/*K*_0_). The value of 1/*K*_0_ is obtained by ion mobility spectrometry mounted on timsTOF as a value of ion mobility, and shows the ion shapes and charge states [6].

**Figure 2:**
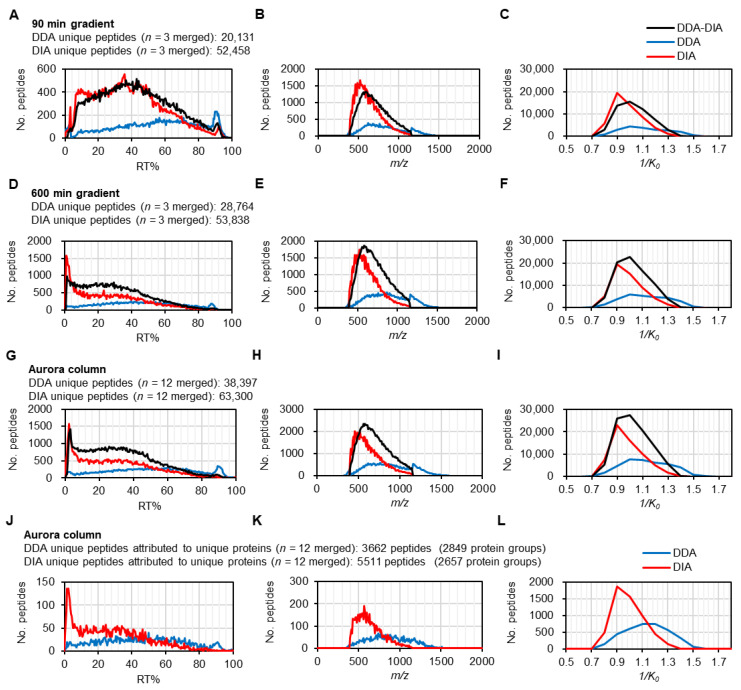
Characteristics of uniquely identified peptides in DDA and DIA methods. (**A**–**C**) Elution profiles of uniquely identified peptides by DDA (*n =* 3) and DIA (*n =* 3) using 90 min analysis with Aurora column across retention-time percent (**A**), *m/z* range (**B**), and 1/*K*_0_ range (**C**). (**D**–**F**) Elution profiles of uniquely identified peptides by DDA (*n =* 3) and DIA (*n =* 3) using 600 min analysis with Aurora column across retention-time percent (**D**), *m/z* range (**E**), and 1/*K*_0_ range (**F**). (**G**–**I**) Elution profiles of uniquely identified peptides by DDA (*n =* 12) and DIA (*n =* 12) with Aurora column across retention-time percent (**G**), *m/z* range (**H**), and 1/*K*_0_ range (**I**). (**J**–**L**) Elution profiles of uniquely identified peptides attributed to unique proteins by DDA (*n =* 12) and DIA (*n =* 12) with Aurora column across retention-time percent (**J**), *m/z* range (**K**), and 1/*K*_0_ range (**L**).

To keep the gradient time, peptide amount, and number of measurements constant for DDA and DIA, we analyzed the results of measurements using 90 min, 600 min gradient times, and all gradient times using the Aurora column (Figure 2). Results from the 90 min analysis showed that while DIA unique peptides were more abundant in the first half of elution time, DDA unique peptides were present throughout the entire elution time, with a slight increase during the latter part of elution time (Figure 2A). When the gradient time increased to 600 min, more DIA unique peptides were identified, especially in the first 10% of elution time (Figure 2D). The analysis of all gradient times using the Aurora column showed a similar distribution to the 600 min analysis (Figure 2G, Table S2), suggesting that the complexity of peptides within the first 10% of elution time was very high and that many peptides were identified specifically by DIA, with longer separation time. The median values of 1/*K*_0_ and *m/z* for DDA unique peptides were 1.0–1.1 and 794–869, respectively, while the median 1/*K*_0_ and *m/z* for DIA unique peptides were 0.9 and 585–592, respectively (Figure 2B,C,E,F,H,I). These results indicate that DIA unique peptides have generally smaller 1/*K*_0_ and *m/z* values, and most of them were detected at the beginning of elution time. In contrast, the unique peptide identification tended to decrease in the latter half of the elution time, especially in DIA. Since the DIA method used in this study was limited to the range of 391–1175 (*m/z*) and 0.69–1.39 (1/*K*_0_) and did not target peptides above 1175 *m/z*, it was assumed that the DDA method has more unique peptides above 1175 *m/z*. The results were partially consistent with this assumption, indicating that the DDA method detected more unique peptides in the higher *m/z* region and that the DIA method was less efficient in identifying unique peptides especially those above 900 *m/z* (Figure 2B,E,H). One possible reason is that the collision energy of MS/MS acquisition by DIA method is not optimal for high *m/z* and 1/*K*_0_ values.

Finally, we examined how the unique peptides with unique proteins were distributed by each method (Figure 2J–L). As with the result at the unique peptide level (Figure 2A–I), DIA unique peptides attributed as unique proteins were present at 400–800 *m/z* and 1/*K*_0_ of 0.8–1.2 during the first half of elution time, but DDA unique peptides were present above 1/*K*_0_ of 1.1 and 900 *m/z* and distributed throughout the elution time. In summary, DIA unique peptides were identified at the beginning of elution time and tended to decrease in the second half. In order to improve the identification efficiency of the DIA method, it is necessary to optimize the liquid chromatography conditions and collision energy during MS/MS acquisition, especially for the peptides above 1/*K*_0_ of 1.1 and 900 *m/z* in the latter half of the elution time.

### 3.3. Characteristics of Phosphorylated- and Ubiquitinated-Proteome Analysis

**Figure 3:**
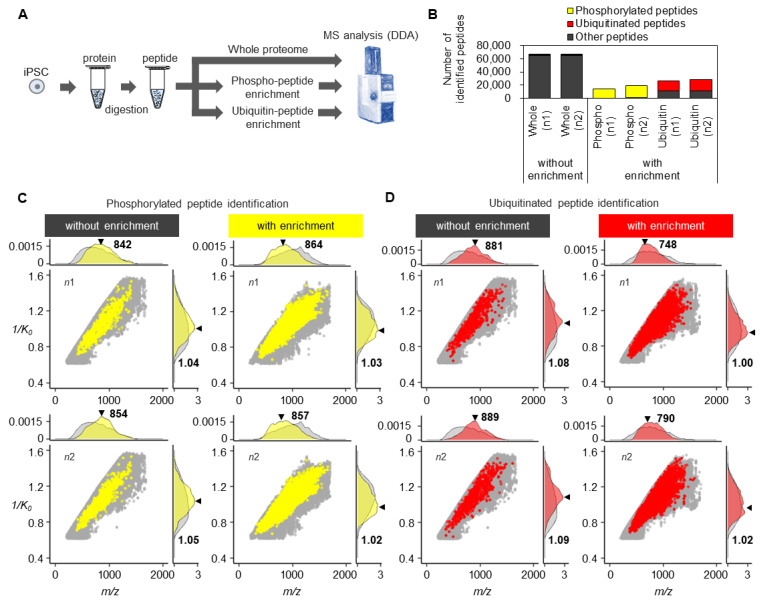
Characteristics of post-translationally enriched peptides. (**A**) Workflow of proteome analysis of whole, phosphorylated, and ubiquitinated peptides from iPSCs. (**B**) Number of identified peptides in the whole, phosphorylated, and ubiquitinated proteomes. (**C**) Dot and density plots of observed (gray) and identified phosphorylated (yellow) peptides in the indicated 1/*K*_0_ and *m/z* ranges, with or without enrichment of phosphorylated peptides. Triangles and bold numbers show median values of *m/z* and 1/*K*_0_. (**D**) Dot and density plots of observed (gray) and identified ubiquitinated (red) peptides in the indicated 1/*K*_0_ and *m/z* ranges, with or without enrichment of ubiquitinated peptides. Triangles and bold numbers show median values of *m/z* and 1/*K*_0_.

So far, we have shown the distribution of the whole proteome, but how about post-translationally modified peptides, such as ubiquitin and phosphorylation modifications? To clarify this, we analyzed whole and enriched ubiquitinated and phosphorylated peptides from human iPSCs (Figure 3A, Table S3). Without enrichment, the number of phosphorylated and ubiquitinated peptides was approximately 500, or 0.8–1% of all identified peptides (Figure 3B, Table S3). However, after enrichment, approximately 16,000 phosphorylated peptides were identified, with 97% enrichment efficiency. Although the enrichment efficiency of ubiquitinated peptides was lower than that of phosphorylated peptides, approximately 17,000 ubiquitinated peptides were identified with 60% enrichment efficiency.

The *m/z* and 1/*K*_0_ distributions of the total, phosphorylated, and ubiquitinated peptides are shown in Figure 3C,D. Phosphorylation-modified peptides had broader distributions of *m/z* and 1/*K*_0_ values than without enrichment, as previously reported [22]. Targeting specific slice regions of *m/z* in the whole proteome, such as with the Thin-PASEF method, improves the peptide identification efficiency by the DIA method [23]. However, setting the target toward wider *m/z* and 1/*K*_0_ ranges should improve the identification efficiency for phosphorylated peptides. Like phosphorylated peptides, the distribution was spread similarly toward wider *m/z* and 1/*K*_0_ ranges for ubiquitin modification. However, unlike phosphorylation, median values of *m/z* and 1/*K*_0_ shifted toward smaller numbers. Therefore, including lower 1/*K*_0_ at lower *m/z* to target the ubiquitin proteome may improve the identification efficiency. Although the current DIA analysis still has difficulty identifying various types of post-translationally modified peptides comprehensively, mainly because of informatics problems, these results suggest that a different mass range to target post-translationally modified peptides would be beneficial for increasing identification efficiency.

## 4. Discussion

Here, we examined how the peptides uniquely detected by DDA and DIA methods differ. Using over 10,000 proteins and peptides identified by each method, we found that approximately 19–32% of peptides were unique and had distinct elution, *m/z*, and 1/*K*_0_ profiles. However, there were no significant differences in amino acid composition, and DIA uniquely identified short peptides, especially those between 7 and 16 amino acids. In particular, the cumulative identification number was higher for DIA, which is a powerful advantage in quantitative analysis. By contrast, the DIA method often uses narrower *m/z* and 1/*K*_0_ ranges than DDA, and excludes the high *m/z* range to reduce the complexity of MS/MS analysis. Many DDA unique peptides in this study were identified during the latter half of elution time, where peptides with relatively high *m/z* were eluted. To further improve the identification efficiency of DIA, it is necessary to develop a method that can change the *m/z* and 1/*K*_0_ ranges according to the elution time. Furthermore, it is essential to separate the peptides during the latter part of elution time and optimize the collision energy of MS/MS acquisition, especially for peptide ions above 900 *m/z* and 1/*K*_0_ above 1.1. Previously, the highest identification efficiency in a single DIA analysis was approximately 10,000 proteins. However, with the recent advances in MS instruments, the Orbitrap Asymmetric Track Lossless (Astral) mass spectrometer with faster scan speeds to keep up with the complexity of the protein sample is now available, making it possible to identify 13,000 proteins in a single DIA analysis [4]. With such active development of MS instruments, a time will come when we no longer need to consider the complexity of proteome samples. A limitation of this work is that we did not verify whether uniquely detected peptides by the DDA and DIA methods were correctly identified. In the future, we intend to investigate whether DDA and DIA’s unique peptide identifications are correct using synthetic peptide analysis.

Furthermore, we showed that the phosphorylated and ubiquitinated proteomes behave differently from the whole proteome, indicating the importance of establishing appropriate ranges for DIA methods. Recently, DIA methods such as synchro-PASEF [24], slicePASEF [25], midiaPASEF [26], and Thin-PASEF [23] have been developed to improve identification efficiency and quantification accuracy. For example, Thin-PASEF improves identification efficiency by targeting narrow *m/z* and 1/*K*_0_ ranges toward a high ion density and reducing the complexity of MS/MS. For the analysis focusing on post-translationally modified peptides, optimized range settings different from those used to analyze the whole proteome may be effective to improve identification efficiency. With the DIA and data analysis methods updated over the years, identification efficiency has improved, even using the same data [27]. Improved informatics for DIA analysis is likely to accelerate. Thus, comparative analyses of the differences between DDA and DIA, as discussed in this study, are essential to develop methods for optimal peptide identification.

## Data Availability

The mass spectrometry data were deposited in the ProteomeXchange Consortium via jPOSTrepo [28] (https://repository.jpostdb.org/, accessed on 19 July 2024) with the dataset identifier JPST003416 (PXD056754).

## Supplementary Materials

The following supporting information can be downloaded at https://www.mdpi.com/article/10.3390/cells13221848/s1, Table S1. The number of protein and peptide identifications shown in Figure 1B,C. Table S2. The list of uniquely identified peptides by DDA and DIA methods using the Aurora column shown in Figure 1 and Figure 2. Table S3. The list of identified peptides from whole, phosphorylated, and ubiquitinated proteomes shown in Figure 3.
